# Radiogenomic analysis of prediction HER2 status in breast cancer by linking ultrasound radiomic feature module with biological functions

**DOI:** 10.1186/s12967-022-03840-7

**Published:** 2023-01-24

**Authors:** Hao Cui, Yue Sun, Dantong Zhao, Xudong Zhang, Hanqing Kong, Nana Hu, Panting Wang, Xiaoxuan Zuo, Wei Fan, Yuan Yao, Baiyang Fu, Jiawei Tian, Meixin Wu, Yue Gao, Shangwei Ning, Lei Zhang

**Affiliations:** 1 Department of Ultrasound Medicine, The Second Affiliated Hospital of Harbin Medical University, Harbin, 150086 Heilongjiang China; 2 College of Bioinformatics Science and Technology, Harbin Medical University, Harbin, 150081 China; 3 Department of Surgery, The Second Affiliated Hospital of Harbin Medical University, Harbin, 150086 Heilongjiang China; 4 Department of Clinical Medicine, The Second Affiliated Hospital of Harbin Medical University, Heilongjiang, 150086 China

**Keywords:** Breast cancer, HER2, Radiogenomics, Radiomics, Ultrasound

## Abstract

**Background:**

Human epidermal growth factor receptor 2 (HER2) overexpressed associated with poor prognosis in breast cancer and HER2 has been defined as a therapeutic target for breast cancer treatment. We aimed to explore the molecular biological information in ultrasound radiomic features (URFs) of HER2-positive breast cancer using radiogenomic analysis. Moreover, a radiomics model was developed to predict the status of HER2 in breast cancer.

**Methods:**

This retrospective study included 489 patients who were diagnosed with breast cancer. URFs were extracted from a radiomics analysis set using PyRadiomics. The correlations between differential URFs and HER2-related genes were calculated using Pearson correlation analysis. Functional enrichment of the identified URFs-correlated HER2 positive-specific genes was performed. Lastly, the radiomics model was developed based on the URF-module mined from auxiliary differential URFs to assess the HER2 status of breast cancer.

**Results:**

Eight differential URFs (*p* < 0.05) were identified among the 86 URFs extracted by Pyradiomics. 25 genes that were found to be the most closely associated with URFs. Then, the relevant biological functions of each differential URF were obtained through functional enrichment analysis. Among them, Zone Entropy is related to immune cell activity, which regulate the generation of calcification in breast cancer. The radiomics model based on the Logistic classifier and URF-module showed good discriminative ability (AUC = 0.80, 95% CI).

**Conclusion:**

We searched for the URFs of HER2-positive breast cancer, and explored the underlying genes and biological functions of these URFs. Furthermore, the radiomics model based on the Logistic classifier and URF-module relatively accurately predicted the HER2 status in breast cancer.

**Supplementary Information:**

The online version contains supplementary material available at 10.1186/s12967-022-03840-7.

## Background

Human epidermal growth factor receptor 2(HER2) is a proto-oncogene that encodes a transmembrane tyrosine kinase growth factor receptor and mainly regulates tumor signal transduction and cell proliferation[1]. Approximately, 15%–20% of all breast cancers are positive for HER2. This is associated with highly aggressive disease and poor prognosis [2]. The prognosis of HER2-positive breast cancer has substantially improved with HER2-targeted therapies [3]. A clinical trial showed that the HER2-blockade agent trastuzumab significantly improved event-free survival (EFS) in HER2-positive breast cancer patients [4]. Furthermore, HER2-targeted therapy decreases tumor burden and increases pathologic complete response (pCR) in HER2-positive breast cancer patients [5–7]. Therefore, the identification of HER2 status helps to select the best individualized treatment strategy for breast cancer patients.

Ultrasound has become a recommended method for the diagnosis and follow-up of breast lesions, as it has the advantages of convenience, economical, universality, real-time dynamics and radiation-free [8, 9]. Meanwhile, ultrasound has a relatively high sensitivity and specificity for the diagnosis of breast cancer, especially in dense breast cancers. A credible association between HER2 status and ultrasound features has been identified. HER2-positive breast cancer has been associated with posterior echogenic enhancement, calcifications, and vascularity [10, 11]. However, few studies have established a radiomic model based to ultrasound images to evaluate HER2 status [12–14].

Radiomics, the extraction and analysis of high-throughput features from medical images, is of great importance for the diagnosis, treatment and prognosis in tumor [15]. Ultrasound radiomic features (URFs) are high-dimensional quantitative features extracted by computer from ultrasound images. Related studies on breast ultrasound, URFs were mainly used to construct a radiomic model to classify breast lesions [16–18]. Valeria et al. successfully established a radiomics model based on URFs and random forest algorithm to distinguish malignant lesions from benign with 0.82 of the AUC [19]. However, the relationship between URFs and HER2 status in breast cancer and the biological information of URFs have not been explored in detail.

Radiogenomics associates radiomic features with genetic phenotypes to reveal radiomics-related biological functions [20, 21]. Public data resources, such as the Gene Expression Omnibus (GEO), Gene Ontology (GO), and Kyoto Encyclopedia of Genes and Genomes (KEGG) provide cancer genomic profiling data to promote cross-disciplinary research including radiogenomic studies [22, 23]. Yeh et al.[24] correlated MRI radiomic features with genomic analyses and showed that the enhanced texture of intratumor heterogeneity is associated with the Janus kinase-signal transducer and activator of transcription signaling pathway, which plays an important role in immune regulation [25]. Most radiogenomic studies have focused on MRI [26–29], rather than ultrasound imaging of HER2-positive breast cancer.

HER2 is a marker of prognosis and therapeutic target. In this study, we aimed to establish a radiomics model based on the Logistic classifier and URF-module to noninvasively predict the status of HER2 in breast cancer. The model would help clinicians to better classify patients for precise therapeutic care. Meanwhile, we explored the potential genes and biological functions of URFs. These discoveries will greatly increase the biologic understanding of URFs. It is expected to promote the development of radiogenomics and provide a novel perspective for the study of breast ultrasound using radiogenomics.

## Materials and methods

### Patients

The requirement for informed consent was waived because of the retrospective study design and the use of images and clinical information about patients derived from medical records. This study included patients who underwent preoperative ultrasound between January 2012 and December 2022 from the Second and the Third Affiliated Hospital of Harbin Medical University.

The patients were included when they met the inclusion criteria: a. pathologically confirmed breast cancer by core needle biopsy or surgical resection; b. ultrasound examination performed before invasive procedures; c. HER2 status of tumors was definite and d. evident lesions on ultrasound images. The exclusion criteria were listed as follows: a. patients who receive any treatment, such as neoadjuvant chemotherapy and radiotherapy, before ultrasound images collection; b. breast cancer of patients with equivocal HER2 status; c. patients with poor image quality; d. patients underwent invasive biopsy, which destroyed the image, before ultrasound images collection; e. patients with incomplete clinical data. Patient enrollment process of the study show in Additional file 1: Fig. S1.

**Fig. 1:**
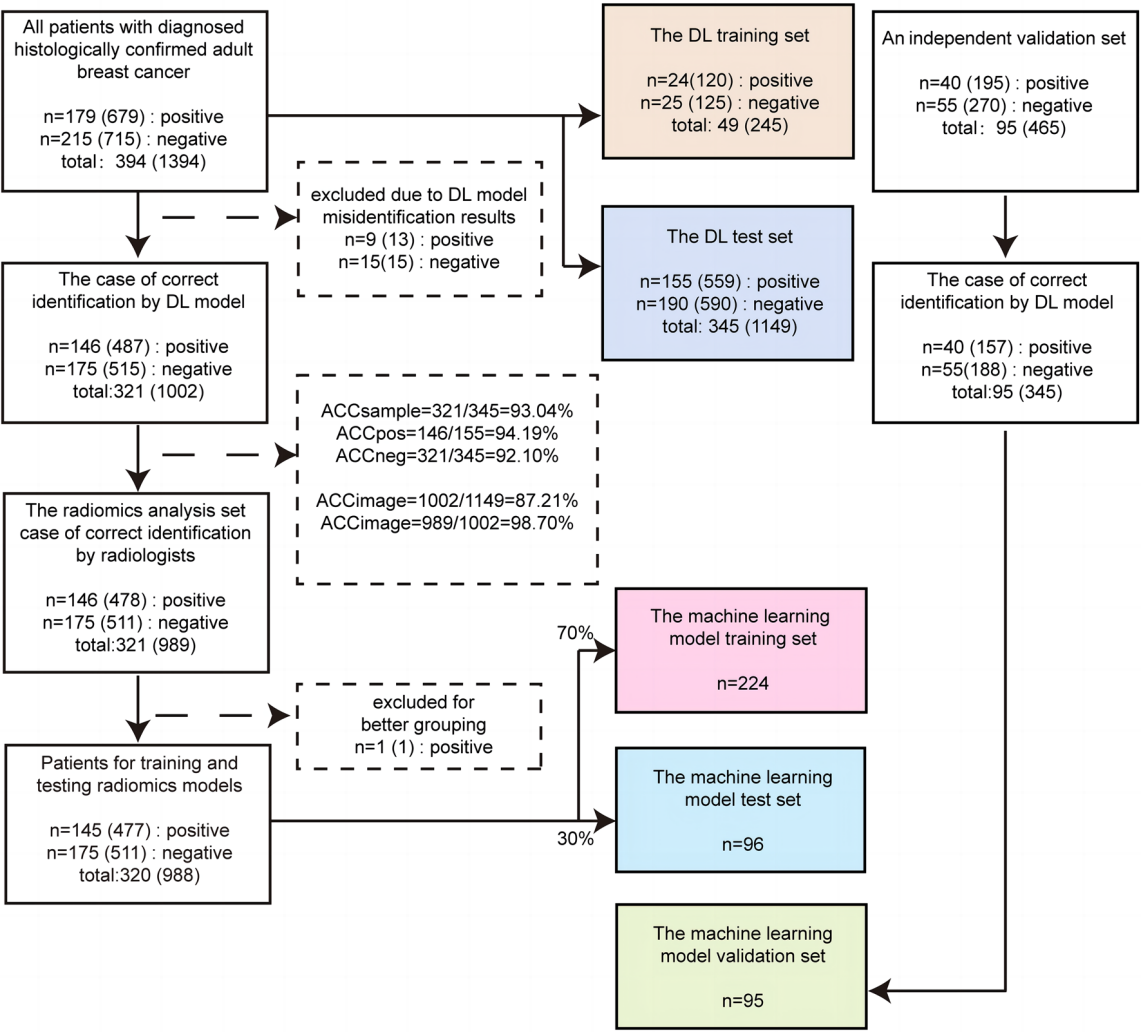
Flow chart of cohort selection. The left panel shows the sample selection process in the training and test sets; the middle panel represents the sample distribution in the model, and the right panel indicates the sample selection process in the independent validation set. *ACC* Accuracy, *DL* Deep Learning

**Fig. 2:**
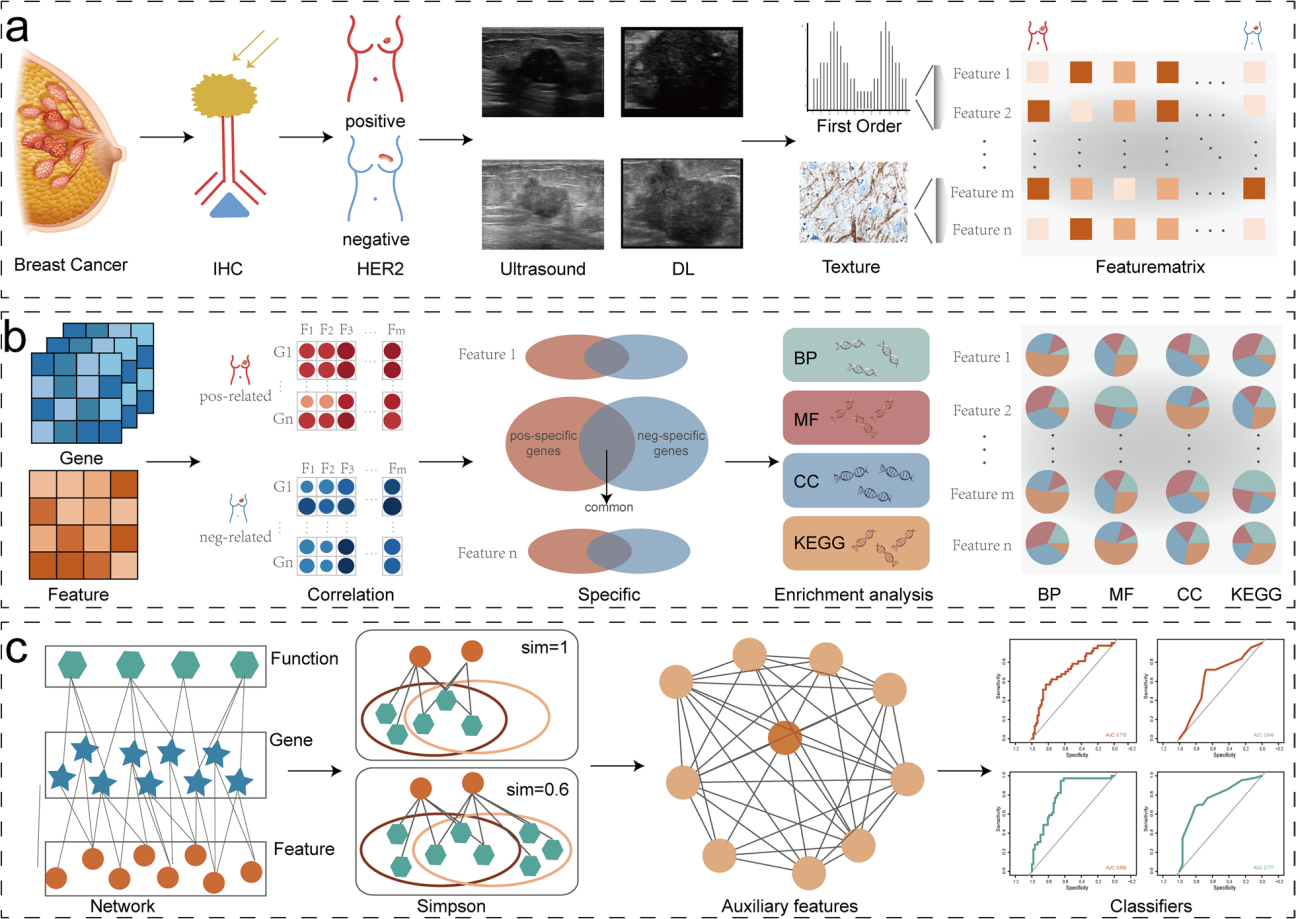
Flow diagram of study. First **a**, positive and negative samples are immunohistochemically distinguished, and tumor regions and radiomic features are extracted using the deep learning model. Second **b**, radiomics features are combined with gene expression data, and the biological functions of radiomic features are inferred by enrichment analysis. Third **c**, auxiliary features are identified based on Simpson index, and machine learning models are constructed

The patient data comprised the following data sets: a. a region of interest (ROI) training set to develop a model that could automatically classify breast cancer/background; b. an ROI test set to evaluate the performance of the ROI classification model; and c. a radiomics analysis set that included breast cancer ultrasound images after accurate segmentation by the ROI classification model and radiologists to extract URFs; d. then we divided the patients from the radiomics analysis set into a radiomics training set and a test set for training and testing the model at a ratio of 7:3; e. finally, an independent validation set is added to further evaluate the performance of the model. The cohort selection flowchart is shown in Fig. 1. The flow diagram of study is shown in Fig. 2.

In this study, 489 breast cancer patients (mean age, 52 years ± 10 [standard deviation]; all women) were finally included. This study contained a total of 1859 ultrasound images, including 874 ultrasound images from 219 HER2-positive breast cancer patients and 985 ultrasound images from 270 HER2-negitive breast cancer patients. And five main data sets were included in this study for analysis: the ROI analysis set contained 49 patients, the ROI test set contained 345 patients, the radiomics training set contained 224 patients, the radiomics test set contained 96 patients and the independent validation set contained 95 patients.

### Assessment of HER2 status

All patients underwent breast cancer surgery or core needle biopsy to obtain tumor tissue for pathological diagnosis, right away after ultrasound images collection. The tumor tissues were stained with hematoxylin–eosin (HE) and performed in formalin-fixed, paraffin-embedded materials. After 5–7 days, the HER2 status of all breast cancer patients was tested using IHC or FISH according to the 2018 guideline recommendations of the American Society of Clinical Oncology and College of American Pathologists [30]. The IHC staining intensity of HER2 was scored as 0, 1 + , 2 + , or 3 + . The IHC staining intensity scored as 3 + was defined as HER2-positive, whereas the IHC staining intensity scored as 0 or 1 + was defined as HER2-negative. The IHC staining intensity scored as 2 + was defined as equivocal and FISH was used to further confirm HER2 status.

### Ultrasound images acquisition

Ultrasound examinations were performed using HITACHI Vision 500 system (Hitachi Medical System, Tokyo, Japan) and Aixplorer ultrasound imaging system (SuperSonic Imagine, SSI, France), both equipped with a linear probe of 5–13 MHz. Radiologist with over five years of experience were selected for image collection and all radiologists were rigorous trained before collecting ultrasound images. They scanned the lesions from multiple angles, and selected several clear ultrasound images to be used for subsequent analysis.

### CNN-based segmentation and URFs extraction of breast cancer

We used Mask-R-Convolutional Neural Network-based architecture for the breast cancer/background classification of ultrasound images. We initially trained the convolutional layer in the Microsoft Common Objects in Context dataset with a learning rate of 0.001 and 1,000 epochs of 100 batches. Then, the deep learning (DL) algorithm was trained using the ROI training set and tested using the ROI test set.

The radiomic features in ROIs were extracted using the Python package, PyRadiomics (version 2.1.0; https://pyradiomics.readthedocs.io/) [31]. Eighty-six features were extracted from ROIs on the ultrasound images, and differences in features between HER2-positive and -negative samples were identified using Wilcoxon tests.

### Identification of specific gene sets associated with HER2-positive status

Three mRNA expression profiles [GSE45827, GSE129559 (median age, 55 years; range, 32–82), GSE162228] for HER2-positive breast cancer were downloaded from the GEO database based on the following inclusion criteria: (a) the organism was Homo sapiens; (b) HER2 status was explicit; (c) the patients were not previously treated. We ensured that the experiments were robust by using three datasets from different research institutions, sequencing platforms, and sequencing methods (high-throughput or array). Since features and genes are different dimensions of data, we applied the formula maximum value normalization as follows:

$${\text{Value}}\_{\text{standardization}}_{{{\text{ij}}}} \, = \,{\text{Value}}_{{\text{i}}} /{\text{max}}({\text{Value}}_{{\text{j}}} )$$

where, Value_i_ is the feature or gene expression value of sample i, and max (Value_j_) is the maximum value of the feature or gene j.

We identified positive-specific gene sets in 75 perturbation studies, each of which contained the same number of randomly selected samples from the URF sets and gene sets of HER2-positive samples. Pearson correlation coefficients (PCC) between the URFs and gene expression in the HER2-positive and HER2-negative samples were calculated to determine the positively- and negatively-related gene sets, respectively, corresponding to each URF. The negative URF-related genes were excluded from the positive URF-related gene sets to obtain positive-specific gene sets.

### Function and similarity of URFs in HER2-positive breast cancer

Functional enrichment of each URF in GO terms (*p* < 0.05) and KEGG pathways (*p* < 0.05) was analyzed using the R package “clusterProfiler” (Version 4.2.2; http://www.bioconductor.org/packages/release/bioc/html/clusterProfiler.html) [32]. We obtained enriched GO terms and KEGG pathways in each URFs. Although some URFs might not be differential, they share similar functions as the differential URFs. We identified such URFs by calculating the Simpson index for each differential URFs. The formula is as follows:

$${\text{sim}}\, = \,{\text{F}}_{{\text{n}}} /{\text{min}}({\text{F}}_{{\text{i}}} ,{\text{F}}_{{\text{j}}} )$$

where F_n_ is the number of functions shared by features i and j, F_i_ is the number of functions in feature i, and F_j_ is the number of functions in feature j.

If the Simpson index of a pair URF was > 0.6, the URF was defined as an auxiliary URF, and the URF-module consisting of auxiliary and differential URFs was defined.

### Validation of URFs functionality in an independent dataset

We downloaded mRNA expression profiles of breast cancer from GEO (GSE81538) as the validation set and normalized the maximum value. We performed 25 perturbation studies on the validation set, and positive-specific gene sets were identified in at least two perturbation studies. The functions associated with each URF in these positive-specific gene sets were validated using functional enrichment analysis.

### Development of machine learning models

We trained seven machine learning models (support vector machine, random forest, decision tree, logistic regression, Naive Bayes, artificial neural network, and K-nearest neighbor) based on eight differential URFs. We used 320 samples from the radiomics analysis set, 70% of it being the training set and 30% as the test set, and perturbed each model 10,000 times. The performance of the models was evaluated using receiver operator characteristics (ROC) curves.

To evaluate whether the URF-module could improve the performance of the classifiers, we retrained the seven models based on the URF-module and plotted ROC curves.

### Evaluating the performance of machine learning models in the independent validation set

The CNN model we developed above was applied to segment 465 images from 95 patients in the independent validation set, and the segmentation results were identified by experts. Qualified ROIs are extracted by applying the Pyradiomics package for URFs, and the mean value was taken for multiple images of a patient. Finally, the performance of the differential URFs and URF-module in the independent validation set were verified in seven classifiers and ROC curves were plotted for evaluation.

## Results

### Patient characteristics

The clinical characteristics of patients are summarized in Tables 1 and 2. To determine whether there were differences of clinical characteristics between HER2-negative and HER2-positive samples, we analyzed seven clinical characteristics (age, T, N and M status, lymph node status, TNM stage and menopausal status) in the test and validation set samples using the Wilcoxon nonparametric test. The results showed that there were no significant differences among the seven clinical characteristics, including menopause. These indicated that clinical characteristics did not effect the differential identification of radiomic features (*p* > 0.05).

**Table 1:** Significance of clinical characteristics in train & test cohort

Clinical characteristics	HER2-Positive breast cancer	HER2-Negative breast cancer	*p*
No. of patients	179	215	
Age	51.8 ± 9.9	52.7 ± 10.7	0.258
Lymph nodes			0.200
Positive	90	122	
Negative	89	92	
T			0.598
T1	75	86	
T2	103	125	
T3	1	4	
N			0.102
N0	108	99	
N1	51	115	
N2	14	1	
N3	6	0	
M			0.102
M0	177	207	
M1	2	8	
TNM stage			0.795
I	53	57	
II	103	144	
II	21	5	
IV	2	9	
Menopausal.status			0.112
Menopausal	83	117	
Premenopausal	96	98

**Table 2:** Significance of clinical characteristics in validation cohort

Clinical characteristics	HER2-Positive breast cancer	HER2-Negative breast cancer	*p*
No. of patients	40	55	
Age	50.5 ± 8.1	51.9 ± 9.6	0.394
Lymph nodes			0.132
Positive	4	12	
Negative	36	43	
T			0.066
T1	8	21	
T2	30	32	
T3	1	2	
T4	1	0	
N			0.052
N0	15	32	
N1	12	11	
N2	10	11	
N3	3	1	
M			NA
M0	40	55	
M1	0	0	
TNM stage			0.083
I	6	15	
II	20	28	
III	14	12	
IV	0	0	
Menopausal.status			0.134
Menopausal	17	34	
Premenopausal	23	21	

### Ultrasound images segmentation and HER2 status-related URFs extraction

**Fig. 3:**
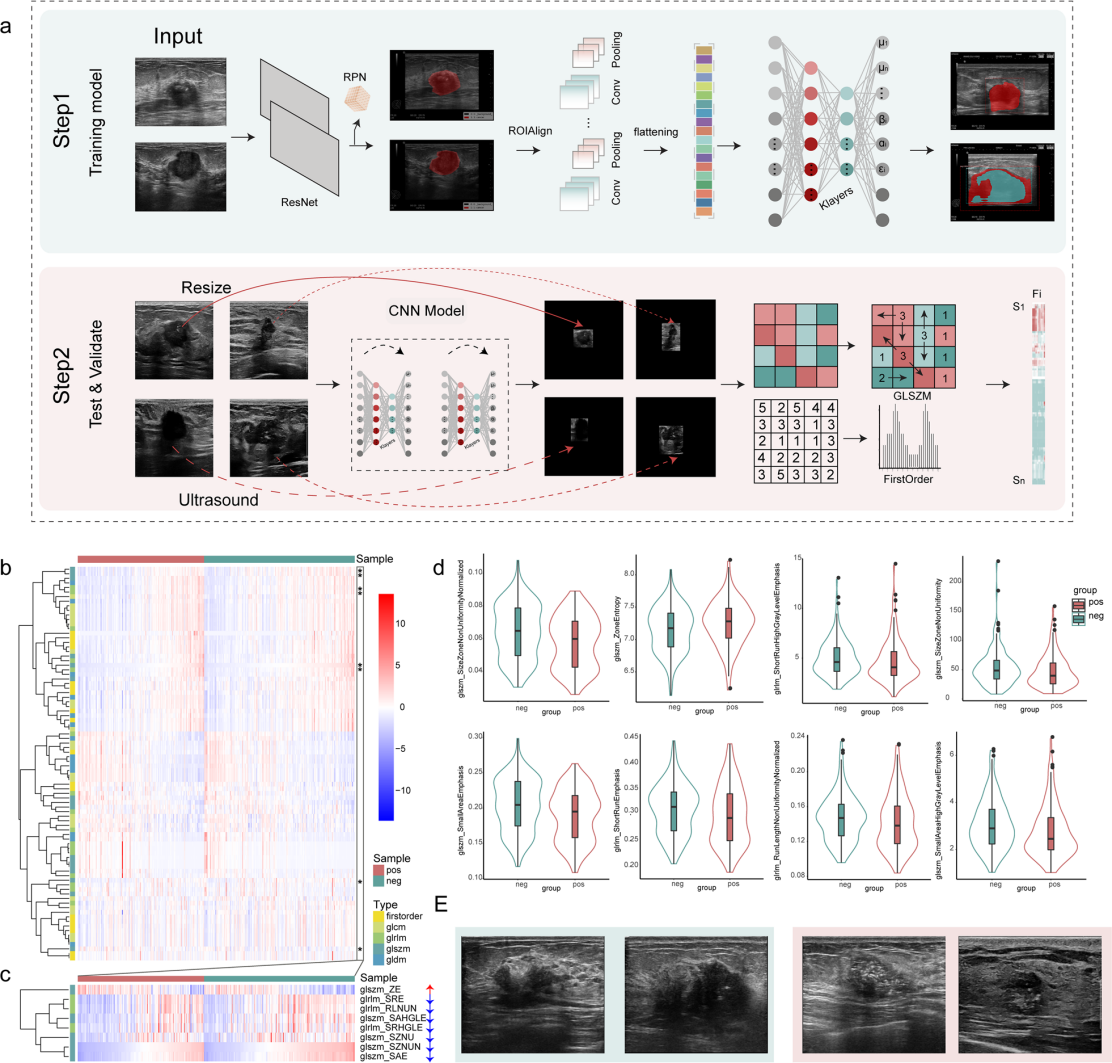
Ultrasound images segmentation and HER2 status-related URFs extraction. **a** Workflow of DL model. First (step 1), training a deep learning segmentation model. Second (step 2), automatically identify tumor regions and extract radiomic features by the model. **b** Heatmap shows all radiomic features between positive and negative samples. Darker shades represent higher levels of features. (*stands for significantly differential feature) **c** Heatmap shows differential radiomic features between positive and negative samples. Violin plots **d** of significantly differential features in negative and positive samples. **e** Ultrasound and immunohistochemical images of patients with negative and positive HER2 status. Green and red, HER2-negative and positive images, respectively

The segmentation model initially identified the tumor region of the breast cancer samples (Fig. 3a), then, 989 of the 1,002 ultrasound images from 321 patients (radiomics analysis set) were accurately segmented with 98.7% accuracy (98.2% and 99.2% for the positive and negative sets, respectively). We then extracted 86 radiomics features (Fig. 3b) for the tumor region, which included First Order Statistics (18 features), Gray Level Co-occurrence Matrix (GLCM; 22 features), Gray Level Dependence Matrix (GLDM; 14 features), Gray Level Run Length Matrix (GLRLM; 16 features), and Gray Level Size Zone Matrix (GLSZM; 16 features).

We analyzed eight differential URFs (Size Zone Non Uniformity Normalized, Zone Entropy, Short Run High Gray Level Emphasis, Size Zone Non Uniformity, Small Area Emphasis, Short Run Emphasis, Run Length Non Uniformity Normalized, Small Area High Gray Level Emphasis) based on the fold-change values (fold change values > 1 and *p* < 0.05, respectively) between the positive and negative samples (Fig. 3c, d). These differential features contained GLSZM and GLRLM categories, indicating different patterns of features in these samples.

To examine whether radiomic features differed among different tumor locations and menopausal status, we tested the URFs of the train & test and validation set patients based on ANOVA (Additional file 1: Figs. S2 and S3). The results showed no significant differences in radiomic features among different tumor location and menopausal status (*p* > 0.05), further indicating that these clinical characteristics did not affect the differential identification of radiomic features.

### Correlation analysis revealed positive-specific gene sets

**Fig. 4:**
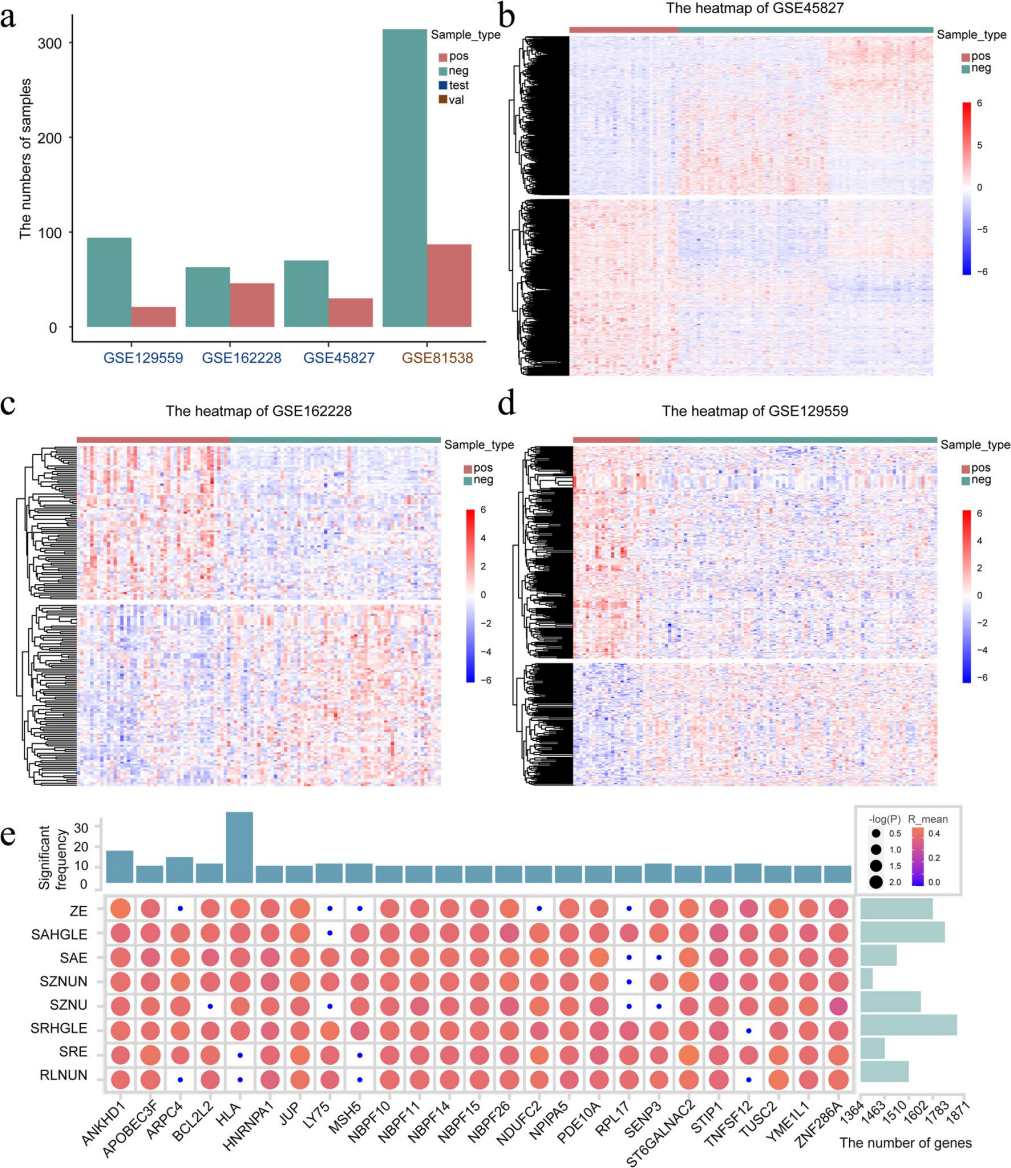
Correlation analysis reveals positive-specific gene sets. Bar chart (**a**), sizes of positive and negative samples in each breast cancer dataset. Heatmaps (**b–d**), gene expression between positive and negative samples in GSE45827, GSE162228, GSE129559. **e** Correlation coefficients between most closely correlated genes and differential features. Higher values and greater significance are shown as more intense red-filled and larger circles, respectively

The three breast cancer mRNA expression profiles of the test set included data from 324 patients (Fig. 4a). The distribution of positive and negative samples did not significantly differ (*p* > 0.05; chi-square test), indicating that it does not lead to biased results; however, the mRNA expression profiles between the two groups differed (Fig. 4b–d). We calculated correlations between differential URFs and genes in each dataset using random perturbation and Pearson correlation analysis (*p* < 0.05, |R|> 0.3). A gene was considered positive-specific only when it significantly associated with positive samples at least six times. We identified a minimum of 1,364 and a maximum of 1,871 positive genes in the eight differential URFs (Fig. 4e). Among the 25 genes with the most associations, we determined that a minimum of 8 and a maximum of 33 were significantly associated (*p* < 0.05). Notably, some URFs were associated with significantly more number of genes. For example, the glszm_Size Zone Non Uniformity(SZNU) feature was associated with 1,871 genes in 75 perturbations and ANKHD1 was significantly associated with all the differential URFs.

### URFs revealed distinct biological functions and similarities

**Fig. 5:**
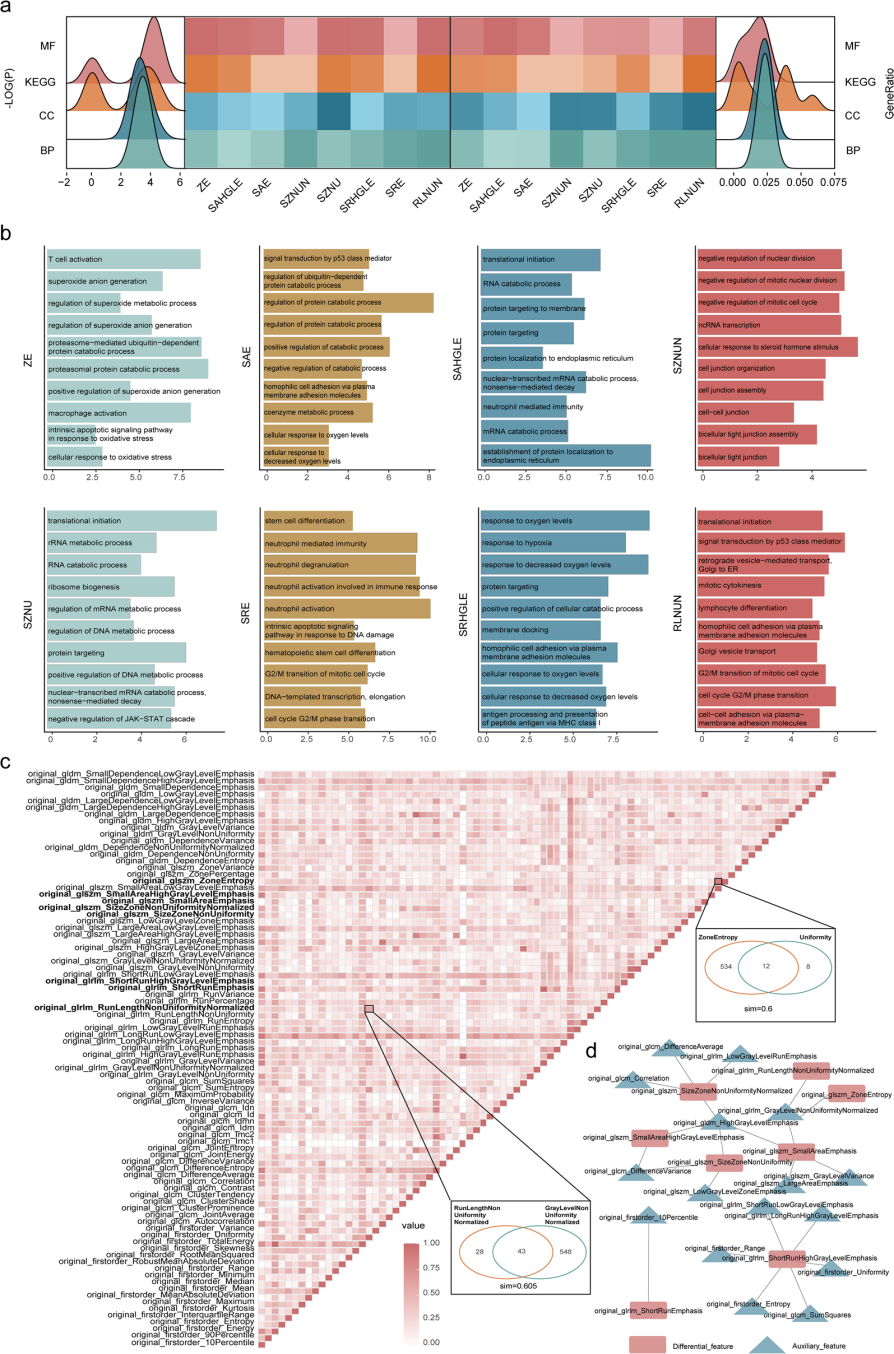
URFs reveals distinct biological functions and similarities. **a** Heatmap of all enriched KEGG pathways and GO terms of differential features. Peaks show distribution of log (p) (left) and gene ratios (right). More intense color represents greater enrichment of features in functions. Bar plots **b** show KEGG pathways and GO terms enriched for specific positive gene sets in each differential feature ranked by -log10(P). **c** Simpson index matrix shows similarity between features of each pair and two examples of feature pairs. Network **d** of the URF-module regulatory relationships

The results of the functional enrichment analysis of the positive-specific gene set (*p* < 0.05) showed that although the significance of differential URFs in the KEGG pathway was lower than that of the molecular function related GO terms, that of the ratio of genes was the highest (Fig. 5a). We then analyzed the functions of the differential URFs, and the results indicated that the Size Zone Non Uniformity Normalized (SZNUN) feature was mainly associated with nuclear division and intercellular communication, Zone Entropy (ZE) participated in immune cell activity and oxidative stress, Short Run High Gray Level Emphasis (SRHGLE) might contribute to cellular hypoxia, Size Zone Non Uniformity (SZNU) affected the catabolism of various compounds, small area emphasis(SAE) was associated with cellular hypoxia and protein metabolism, short run emphasis(SRE) regulated the cell cycle and affected neutrophil activity, Run Length Non Uniformity Normalized (RLNUN) was associated with the cell cycle and intercellular communication, and Small Area High Gray Level Emphasis (SAHGLE) was associated with endosomal localization and structure (Fig. 5b). We found a minimum of one and a maximum of six auxiliary features for each differential URF (Simpson index > 0. 6). Although most of the URFs were specific, some shared similar functions (Fig. 5c). We constructed a regulatory network for the URF-module (Fig. 5d), and the results confirmed known relationships and revealed novel relationships among these URFs.

### Feature functionality was verified in an independent dataset

**Fig. 6:**
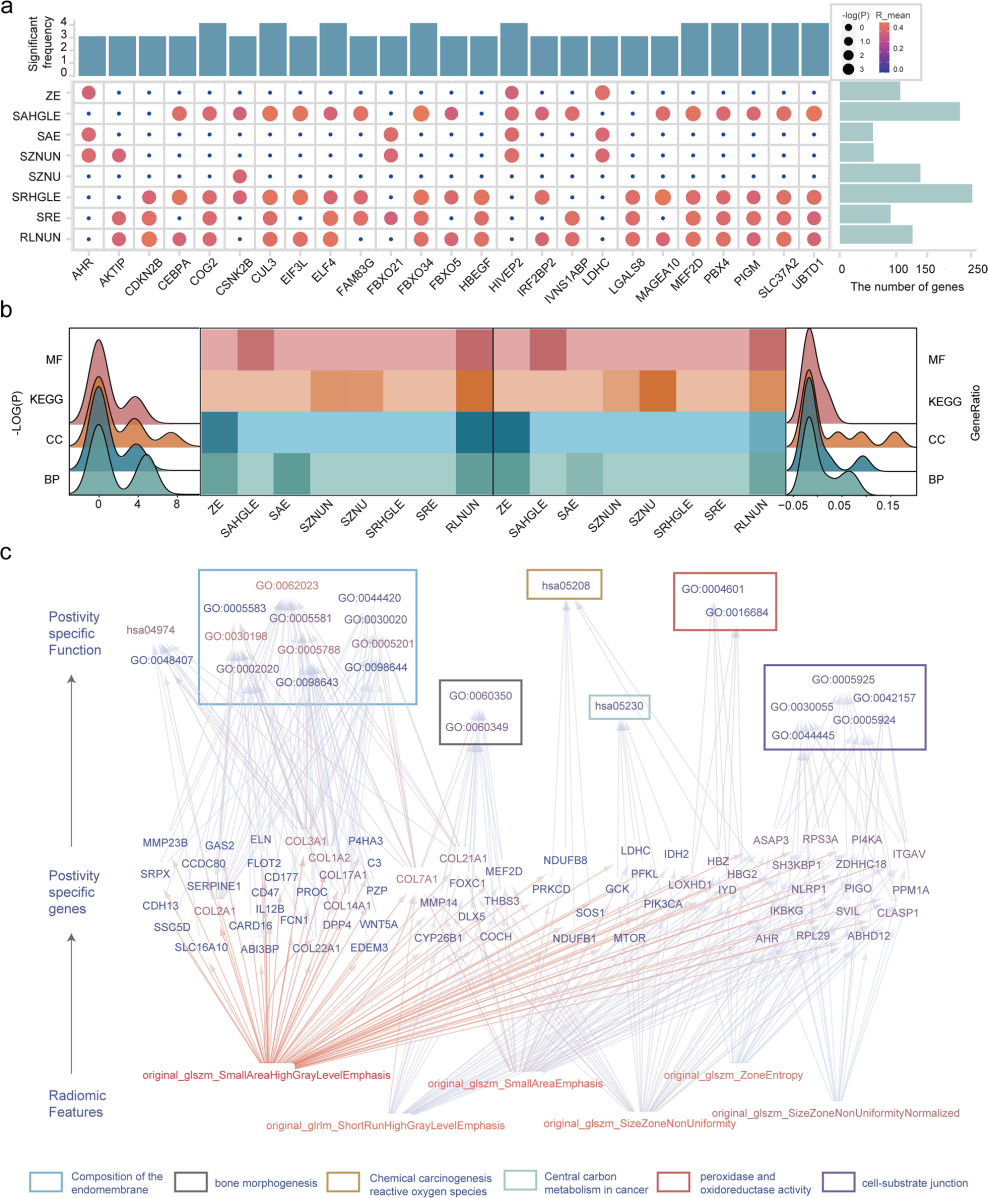
Feature functionality is verified in an independent dataset. **a** Correlation coefficients of most closely correlated genes and differential-features. **b** Heatmap shows all enriched KEGG pathways, GO terms of differential features. Peaks plot show distribution of -log(p) (left) and generation (right). **c** Summary of hierarchical model to systematically understand feature-mRNA-function network of HER2 positive breast cancer

The mRNA expression data, GSE81538, included 314 negative and 87 positive samples. To verify the functions of differential URFs, we identified a minimum of 62 and a maximum of 248 positive-specific genes among the eight differential URFs using Pearson correlation analysis and perturbation studies *(p* < 0.05, |R|> 0.3) (Fig. 6a). Among the 25 genes with the highest number of associations, we determined a minimum of 3 and a maximum of 4 significant associations. The results of analyzing the functional enrichment of the positive-specific gene sets (*p*.adjust < 0.05) showed that the most significant genes and the highest ratio of genes in KEGG pathways associated with the differential URFs and the second most significant genes in the GO terms were related to biological processes (Fig. 6b).

The functions of five differential URFs (fold change values > 1 and *p* < 0.05, respectively) were validated, and the results revealed that SZNU was associated with carbon metabolism in cancer, SZNUN was associated with cell-substrate junction, SAE is associated with chemical carcinogenesis - reactive oxygen species, SAHGLE was associated with the composition of the endomembrane, and ZE was involved in peroxidase and oxidoreductase activity (Fig. 6c).

### URF-module contributed to classification of HER2-positive breast cancer in multiple classifiers

**Fig. 7:**
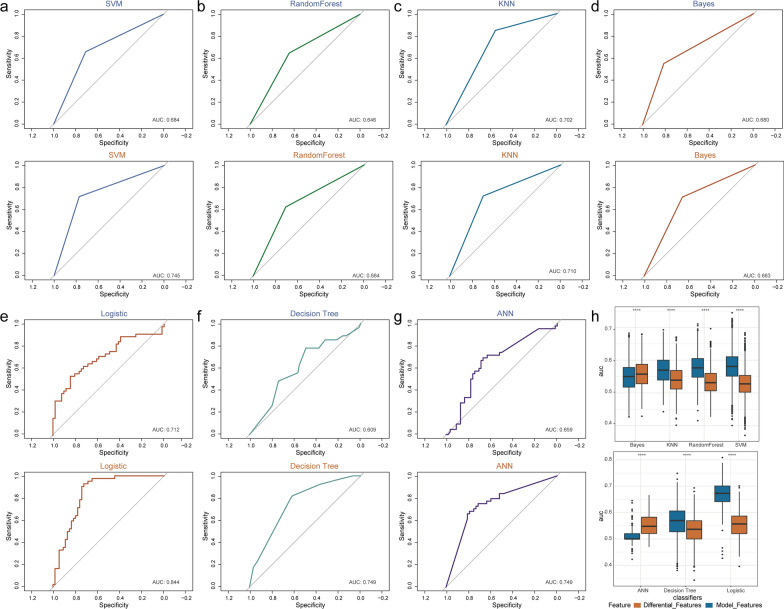
URF-module contributes to classification of HER2-positive breast cancer in multiple classifiers. **a**–**g** Receiver operator characteristics (ROC) curves for seven classifiers. Top and bottom, based on difference URFs and URF-module, respectively. **h** Box plot represents area under the ROC curves for the classifiers based on differential URFs and URF-module

To evaluate the ability of URFs to identify HER2 status, we first trained seven machine learning classifiers, including support vector machine, logistic, Bayes, Decision-Tree, random-forest, artificial neural network, and the K Nearest Neighbor algorithm based on the eight differential URFs (Fig. 7a–g). We then plotted ROC curves to facilitate and analyze comparisons among the different classifiers by measuring areas under ROC curves (AUC). An AUC of 1.0 indicated that the tested classifier was suitable for our data, whereas 0.5 indicated that the classifier had no classification capability for our data. The range of AUC of the tested classifiers was 0.62–0.715, suggesting that the logistic classifier achieved relative accurately classifying HER2-positive breast cancer patients.

We further evaluated the performance of the classifiers based on the URF-module. It can be found that the overall performance of the URF-module based model were better than those of URFs based model (Additional file 1: Table S1). In particular, the specificity of URF-module based model was 80.8% using logistic classifier in test set, implying a low rate of false positive. These results indicated that the URF-module were meaningful for identifying patients who are HER2-positive breast cancer and that the classification accuracy and specificity were improved (Fig. 7h).

### URF-module in independent validation sets contribute to the classification of HER2-positive breast cancer

**Fig. 8:**
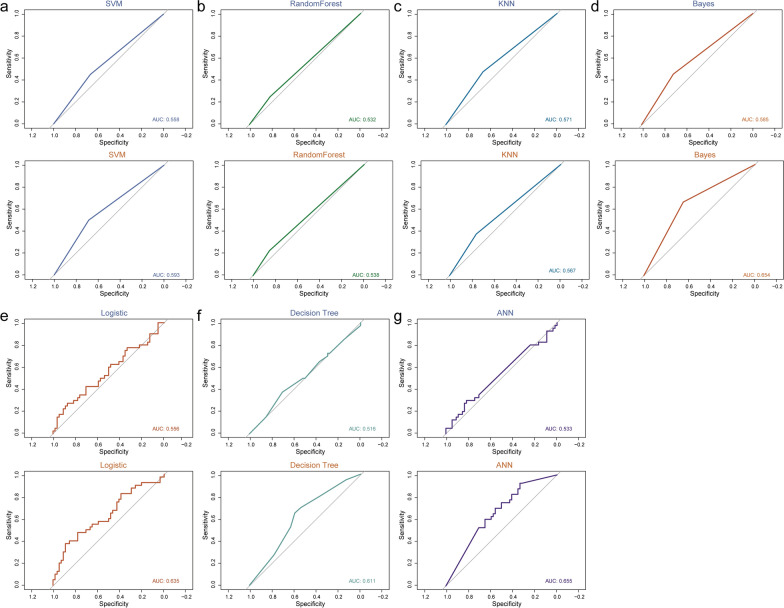
URF-module in independent validation set contributes to the classification of HER2-positive breast cancer. **a**–**g** ROC curves for seven classifiers in validation set, top based on difference features, bottom based on URF-module

To validate the ability of URFs to identify HER2-positive breast cancers, we evaluated the features extracted from the independent validation set based on the seven optimal classifiers. For each classifier, the AUC values ranged from 0.516 to 0.585. We also validated the performance of the URF-module based classifier with AUC values between 0.538 and 0.655(Fig. 8). Similar to test set, the overall performance of the URF-module based model were better than those of URFs based model (Additional file 1: Table S1). In particular, the specificity of URF-module based model was 83.3% using logistic classifier in independent validation set, also mean that a low rate of false positive. These results indicated that the URF-module can improve the classifier's ability to classify HER2-positive breast cancer patients.

## Discussion

The prognosis for patients with HER2-positive breast cancer considerably improved after the advent of HER2-targeted therapies [33]. Therefore, it is important to assess HER2 status for targeted therapy selection. We developed a radiomics model with URF-module based on two-dimensional ultrasound images that could evaluate HER2 status. In addition, we deeply mine URFs of HER2-positive breast cancer to obtain the molecular biological information inherent in URFs of HER2-positive breast cancer.

Eight differential URFs assigned to two types of texture features (GLSZM, GLRLM), were significantly associated with HER2-positive breast cancer. One of the differential URFs was ZE, which we used to measure uncertainty in the distribution of zone sizes and gray levels. A higher ZE value indicated more heterogeneous voxel intensity in the ultrasound images. ZE alone showed an upward trend, whereas the rest of the URFs showed a downward trend. The intrinsic definitions of the other URFs implied that these texture features were associated with not only the uniformity of size zone volumes or run lengths, but also to the relationship between short runs or small areas and high gray level values. These findings showed that the eight differential URFs of HER2-positive breast cancer were characterized by high heterogeneity and low gray levels. This implied that HER2-positive breast cancer often exhibit characteristic calcifications and hypoechoic regions in ultrasound images. These results agreed with those of previous studies [10, 34, 35]. In future work, predicting the status of HER2 in some cancer such as ovarian cancer maybe could be evaluated by this algorithm.

ZE was an important URF showing an upward trend in ultrasound images of HER2-positive breast cancer. In addition, calcification is prevalent in HER2-positive breast cancer [36, 37]. Functional enrichment analysis revealed that ZE associated with immune cell activity, and others confirmed that epithelial cells with a mesenchymal phenotype can assume an osteoblast-like phenotype and undergo complex forms of calcification after BMP-2 induction. This suggested that altered immune cell activity leads to an increase in BMP-2, eventually resulting in the occurrence of calcification in breast cancer [38, 39]. Moreover, functional enrichment analysis also associated ZE with oxidative stress, which might trigger vascular calcification. Further investigation is needed to determine whether oxidative stress can also trigger calcification in breast cancer [40].

We developed seven classifiers to evaluate the ability of URFs, out of which support vector machine, decision tree, and random forest can solve high-dimensional problems, whereas support vector machine can improve generalization; however, a general solution for nonlinear problems is not available. The decision tree has a short run time, but correlations among features are easily ignored. Random forest is more resistant to overfitting, but might not produce good results for small-scale data. Bayes requires few parameters, but the error rate is high because prior probabilities need to be calculated. The K Nearest Neighbor algorithm is more suitable for automatic classification with a relatively large sample size, but the interpretability of the results is low. Lastly, artificial neural network provides high classification accuracy and robustness against noisy nerves. However, neural networks require numerous parameters and the results are difficult to interpret, which affects credibility and acceptability. Logistic regression resists noisy interference and prevents overfitting using regularization, but it is limited by the hypothesis of linearity between features and targets. We found that the logistic classifier produced the largest AUC, indicating a linear association between radiomics features and HER2 status.

We created URF-module to improve the performance of the model. Firstly, considering ten positive Events per Variable (tenfold EPV) regression analysis is widely accepted and the maximum number of variables in the 147 positive samples in this study was 14, we chose 8 URFs for the establishment of the model. However, the results showed that the model was underfitted. Then, we considered the value of event of independent variable (EIV); other studies have reported that Type I error can be better controlled by the PLE-based contour method and maximum likelihood-based Wald method when the EIV is > 20. Therefore, we added auxiliary features to construct a URF-module, and the AUC for classification was significantly increased.

This study had a few limitations. Firstly, the retrospective selection of patients might have led to a selection bias. Therefore, future prospective studies are needed to verify our results. Secondly, our patient cohort was relatively small. More patients should be included in further studies to improve the performance of our model. Finally, we used only two-dimensional ultrasound images. We plan to focus on extracting more image information from multimodal ultrasound images to further understand the link between ultrasound phenotypes and the molecular mechanisms of breast cancer. In addition, biological experiments and double-blind datasets should be performed to validate the computational algorithm in future work.

## Conclusion

In conclusion, we constructed a radiomics model based on the Logistic classifier and URF-module to identify the HER2 status in breast cancer. We also explored the underlying gene expression and biological information of URFs through  radiogenomic analysis. Significantly, ZE associated with calcification in ultrasound images of HER2-positive breast cancer and can reflect immune cell activity, which also can regulate the formation of calcification in breast cancer.

## Supplementary Information

**Additional file 1. Fig. S1.** Patient enrollment process of the study. **Fig. S2.** The URFs data for subjects from the test set. **Fig. S3.** The URFs data for subjects from the validation set. **Fig. S4.** Recognition results of deep learning model for ultrasound. **Fig. S5.** Examples of ultrasound and immunohistochemical images of 10 patients with breast cancer. **Table S1.** Predictive performance of logistic-based radiomics model for predicting the HER2 status in breast cancer.

## Data Availability

The imaging data and code that support the findings of this study are available from the corresponding author upon request.

## Abbreviations

ACC: Accuracy
HER2: Human epidermal growth factor receptor 2
URFs: Ultrasound radiomic features
AUC: Area under the curve
GEO: Gene Expression Omnibus
GO: Gene Ontology
KEGG: Kyoto Encyclopedia of Genes and Genomes
HMU: Harbin Medical University
IHC: Immunohistochemistry
ROI: Region of interest
DL: Deep learning
PCC: Pearson correlation coefficients
ROC: Receiver operator characteristics
GLCM: Gray Level Co-occurrence Matrix
GLDM: Gray Level Dependence Matrix
GLRLM: Gray Level Run Length Matrix
GLSZM: Gray Level Size Zone Matrix
SZNU: Size Zone Non Uniformity
SZNUN: Size Zone Non Uniformity Normalized
ZE: Zone Entropy
SRHGLE: Short Run High Gray Level Emphasis
SAE: Small area emphasis
SRE: Short run emphasis
RLNUN: Run Length Non Uniformity Normalized
SAHGLE: Small Area High Gray Level Emphasis
