# Supplementary material for: Radiogenomic analysis of prediction HER2 status in breast cancer by linking ultrasound radiomic feature module with biological functions
Source: J Transl Med. 2023 Jan 24;21:44. doi: 10.1186/s12967-022-03840-7 (PMC9875533; doi:10.1186/s12967-022-03840-7)
Supplement: Supplementary file 1 — Additional file 1. Fig. S1. Patient enrollment process of the study. Fig. S2. The URFs data for subjects from the test set. Fig. S3. The URFs data for subjects from the validation set. Fig. S4. Recognition results of deep learning model for ultrasound. Fig. S5. Examples of ultrasound and immunohistochemical images of 10 patients with breast cancer. Table S1. Predictive performance of logistic-based radiomics model for predicting the HER2 status in breast cancer. [file 12967_2022_3840_MOESM1_ESM.pdf]

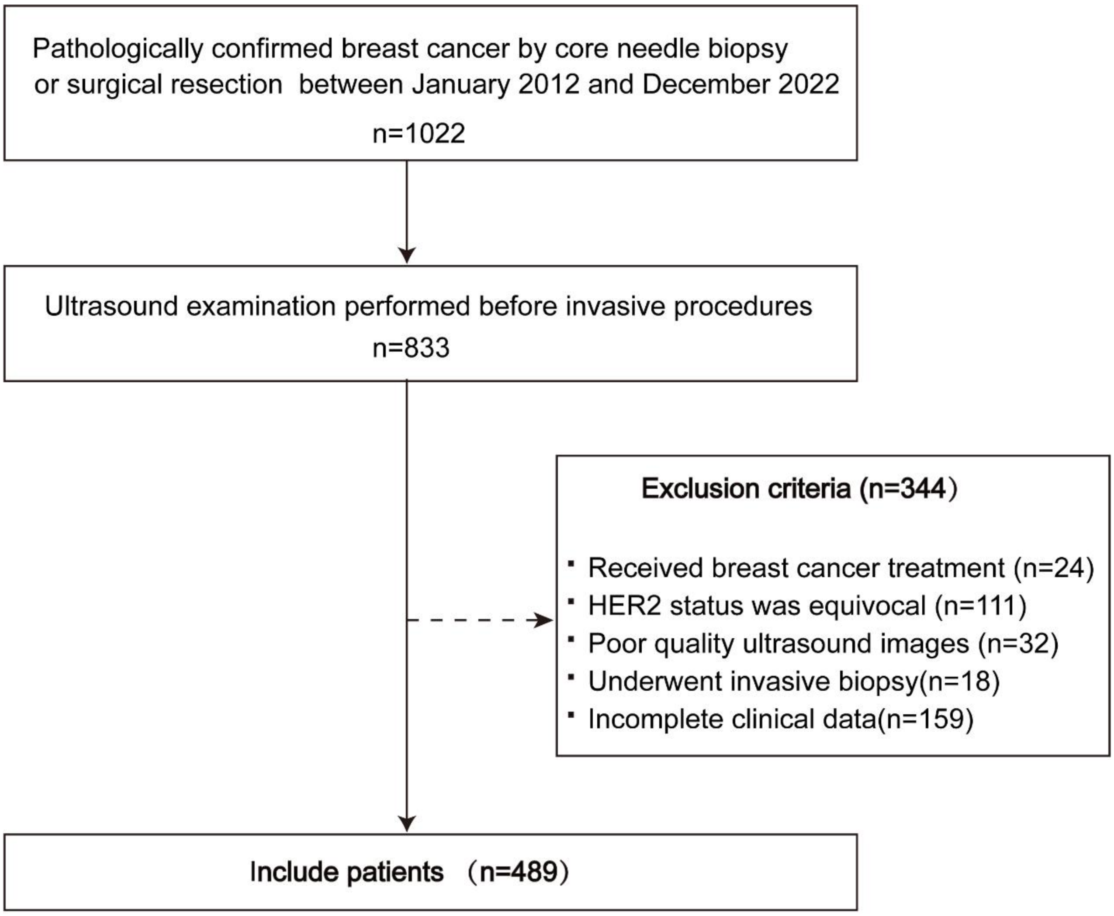

Fig. S1 Patient enrollment process of the study.

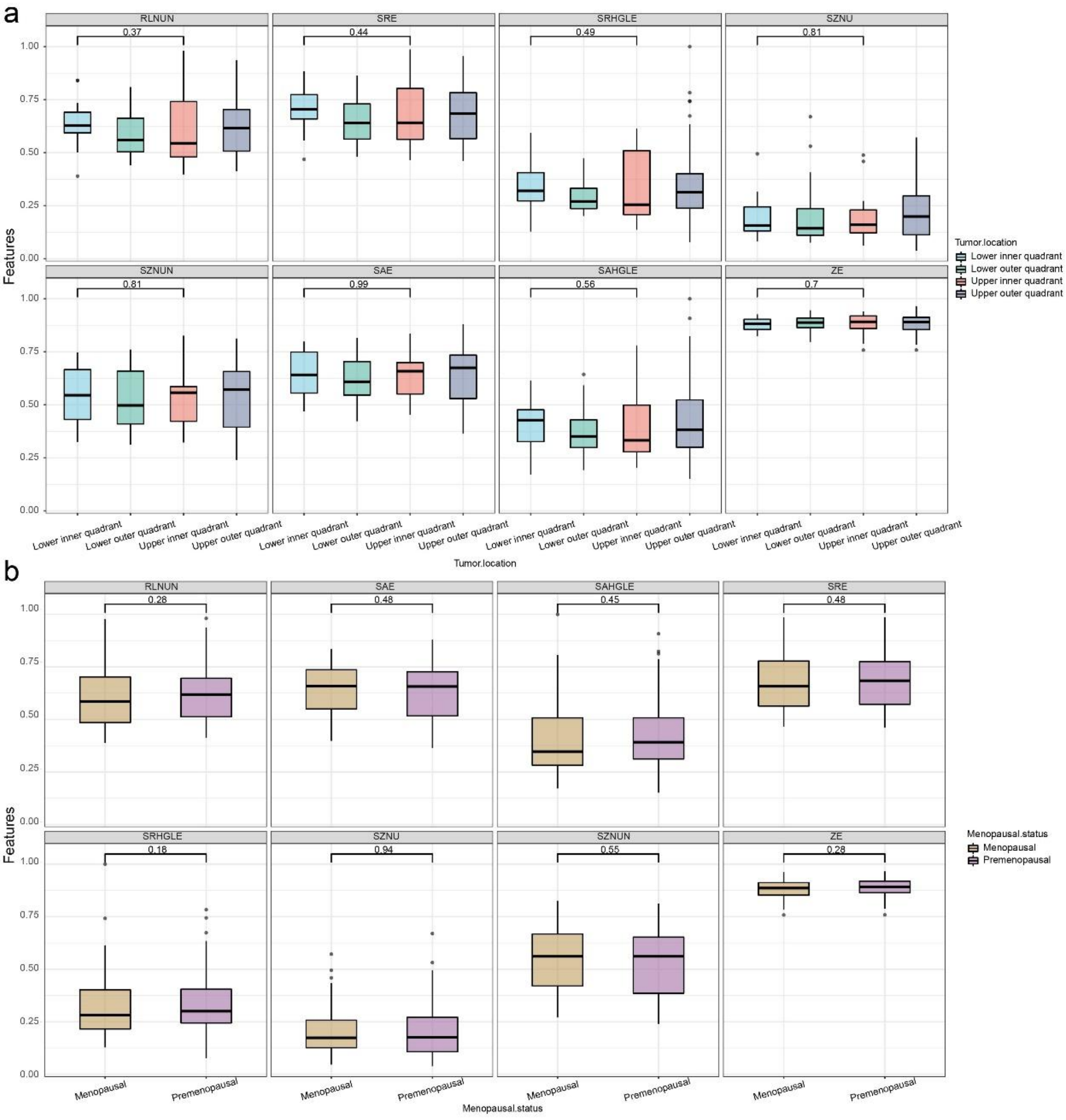

Fig. S2 The URFs data for subjects from the test set.

(**a**) shows four tumor location difference data for eight URFs and (**b**) shows menopausal status difference data. P-values for ANOVA are one-way analyses across sites or menopausal status for each phenotype.

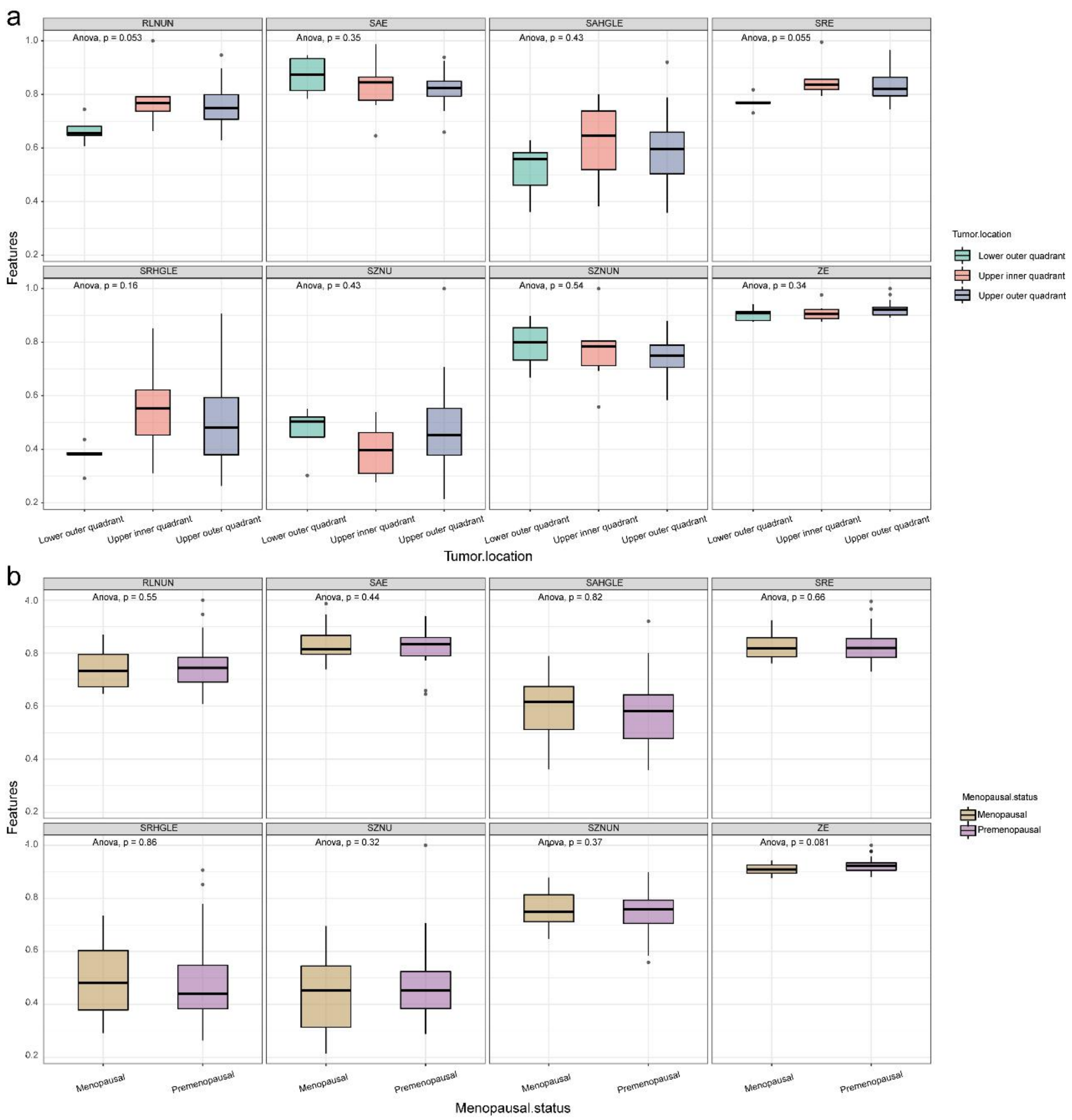

Fig. S3 The URFs data for subjects from the validation set.

(**a**) shows four tumor location difference data for eight URFs and (**b**) shows menopausal status difference data. P-values for ANOVA are one-way analyses across sites or menopausal status for each phenotype.

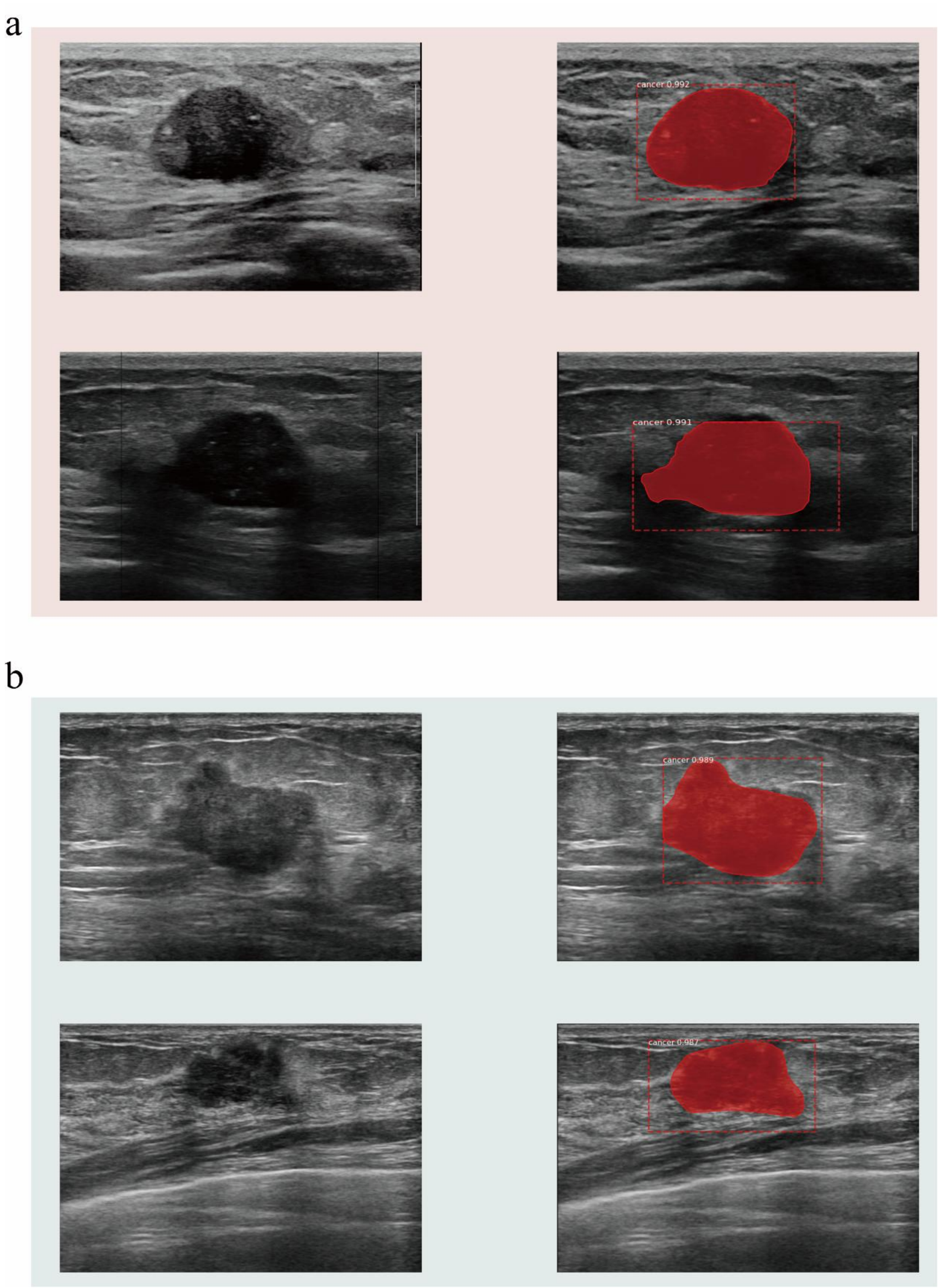

Fig. S4 Recognition results of deep learning model for ultrasound.

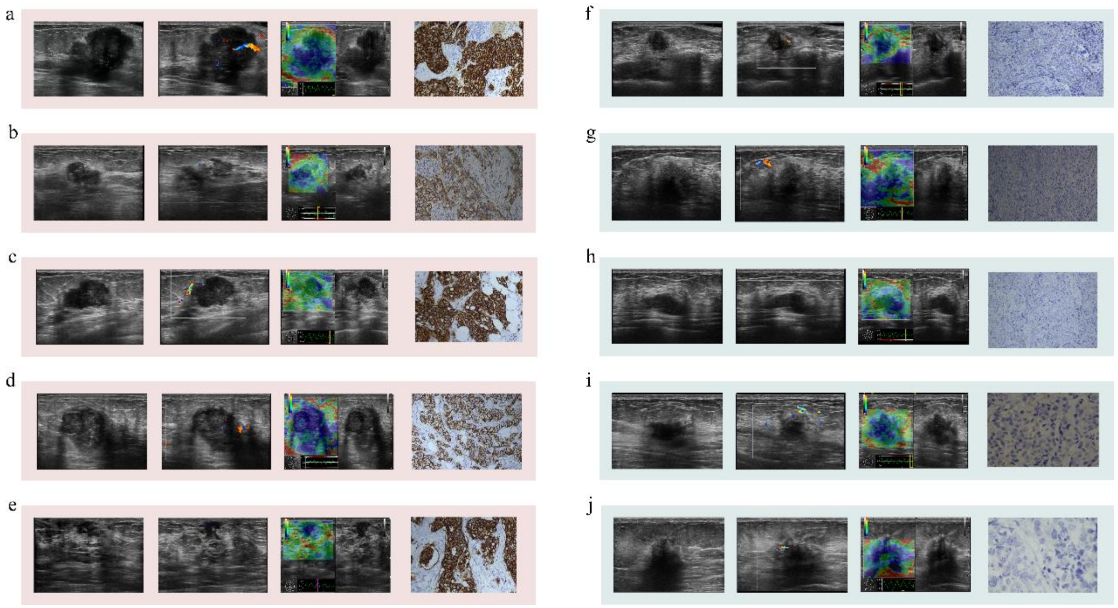

Fig. S5 Examples of ultrasound and immunohistochemical images of 10 patients with breast cancer.

(**a-e**) Two-dimensional, color Doppler, elastic images and immunohistochemical images of 5 cases with HER2-positive. (**f-j**) Two-dimensional, color Doppler, elastic images and immunohistochemical images of 5 cases with HER2-negative.

Table S1: Predictive performance of logistic-based radiomics model for predicting the HER2 status in breast cancer.

	Radiomics test set		Independent validation set	
	**URFs**	**URF-module**	**URFs**	**URF-module**
AUC	0.712(0.620-0.817)	0.844(0.762-0.927)	0.556(0.427-0.665)	0.635(0.511-0.748)
ACC(%)	72.9(72.5-73.3)	80.2(79.9-80.5)	57.4(56.9-58.0)	66.0(65.5-66.4)
SPE(%)	74.5(63.0-86.1)	80.8(70.1-91.5)	64.8(52.1-77.6)	83.3(73.4-93.3)
PPV(%)	67.4(53.4-81.4)	77.8(65.6-89.9)	50.0(34.1-65.9)	65.4(47.1-83.7)
NPV(%)	77.4(66.1-88.6)	82.4(71.9-92.8)	62.5(49.8-75.2)	66.2(54.9-77.4)

Abbreviations: AUC, area under the receiver operating characteristic curve; ACC, accuracy; SPE, specificity; PPV, positive predictive value; NPV, negative predictive value; URFs, ultrasound radiomic features.
